# Clinical Characteristics of Multiple Erythema Migrans in Children: A Five-Year Retrospective Study

**DOI:** 10.3390/pathogens15101037

**Published:** 2026-10-01

**Authors:** Urška Šivic, Mojca Rožič, Franc Strle

**Affiliations:** 1 Department of Infectious Diseases, University Medical Centre Ljubljana, Japljeva 2, 1525 Ljubljana, Slovenia; mojca.rozic@kclj.si (M.R.); franc.strle@kclj.si (F.S.); 2 Faculty of Medicine, University of Ljubljana, Vrazov trg 2, 1000 Ljubljana, Slovenia

**Keywords:** Lyme borreliosis, children, multiple erythema migrans, Lyme neuroborreliosis, clinical presentation

## Abstract

Multiple erythema migrans (MEM) is an early disseminated manifestation of Lyme borreliosis. Data describing its clinical presentation in children are limited, and treatment approaches differ between settings. We retrospectively reviewed children younger than 18 years diagnosed with MEM at the University Medical Centre Ljubljana, Slovenia, from 2021 through 2025. Demographic, clinical, laboratory, treatment, and outcome data were analyzed descriptively. Among 621 children with disseminated Lyme borreliosis, 445 (72%) had MEM. The median age was 5.0 years, and 78% were younger than 8 years. The median number of lesions was 4, and additional lesions usually appeared within a few days of the first lesion. Constitutional symptoms were reported in 28% of children, and routine laboratory findings were largely within age-adjusted reference ranges. Extracutaneous manifestations were uncommon. Most children (95%) received intravenous ceftriaxone. Pediatric MEM predominantly affected young children and usually followed an uncomplicated clinical course characterized by rapid development of additional skin lesions, limited constitutional symptoms, and largely unremarkable routine laboratory findings. Because almost all children received ceftriaxone, this retrospective study cannot compare treatment strategies. Prospective studies are needed to define the optimal antibiotic regimen for pediatric MEM.

## 1. Introduction

Lyme borreliosis (LB) is a tick-borne disease caused by spirochetes of the *Borrelia burgdorferi* sensu lato complex. Slovenia has one of the highest incidences of LB in Europe [1], with a 10-year average incidence (2016–2025) of 216.6 cases per 100,000 inhabitants [2]. Among children aged 1–4 years, the 10-year average incidence is 318 cases per 100,000 inhabitants, the third highest of all age groups; in the last 3 years, this age group had the highest incidence [2].

Erythema migrans (EM) is the characteristic manifestation of early localized LB. It typically begins as a macule or papule that gradually expands over several days to weeks into a red or bluish-red annular lesion, with or without central clearing [3,4]. The presence of two or more EM lesions defines multiple erythema migrans (MEM), a manifestation of early disseminated LB. Although transient constitutional symptoms may accompany or precede the skin lesions [5], patients with MEM generally do not have clinically evident extracutaneous involvement.

Despite the high incidence of LB, studies specifically addressing pediatric MEM remain scarce. A recent Swiss study of 469 children with LB described demographic, clinical, and serological characteristics across manifestations but included only 11 children with MEM [6]. An Austrian study of 55 children with MEM reported younger age compared with solitary EM, male predominance, skin-lesion characteristics, and accompanying extracutaneous symptoms [7]. Apart from a Slovenian study of CSF findings in 214 children with MEM [8], few additional data on the clinical presentation of pediatric MEM have been published.

In Slovenia, intravenous ceftriaxone became standard treatment for pediatric MEM after a 2002 study from our centre reported CSF abnormalities in some neurologically asymptomatic children [8]. Routine lumbar puncture was subsequently abandoned because its findings were not expected to alter treatment. In contrast, current European [5,9,10,11,12] and North American [13] guidelines recommend treating MEM without neurological manifestations with oral antibiotics, as for solitary EM. This difference in practice provides additional rationale for contemporary characterization of pediatric MEM and for future comparative treatment studies.

The diagnosis of typical EM is clinical, but the differential diagnosis is broader when lesions are small or their growth is incompletely documented. Annular lesions in children include inflammatory reactions and other infectious and noninfectious dermatoses, so the evolving morphology, distribution, and clinical context must be considered together [14]. The absence of biopsy confirmation in a retrospective cohort makes subsequent clinical reassessment relevant, although resolution during antibiotic treatment alone does not prove the original diagnosis.

Slovenia is endemic for other tick-borne infections. In 2024, national surveillance recorded 3162 cases of LB and 90 cases of tick-borne encephalitis (TBE) [15]. These figures describe the national epidemiological setting rather than coinfection in children with MEM. Fever and headache around the time of EM may also be nonspecific; microbiological assessment for another tick-borne disease is guided by the clinical presentation rather than performed routinely for every typical EM lesion.

In this retrospective study, we aimed to characterize the clinical presentation of pediatric MEM in a large contemporary cohort, including age and seasonal distribution, timing and distribution of skin lesions, constitutional symptoms, laboratory findings, and clinically apparent extracutaneous manifestations.

## 2. Materials and Methods

### 2.1. Study Design and Patients

This retrospective observational study was conducted at the Department of Infectious Diseases, University Medical Centre Ljubljana, Slovenia. The institutional clinical database was searched for all patients younger than 18 years diagnosed with Lyme borreliosis (ICD-10 code A69.2) between 1 January 2021 and 31 December 2025. Children diagnosed with MEM were included in the study. We selected the five most recent complete calendar years available to describe current clinical practice and annual and seasonal patterns.

MEM was diagnosed clinically according to the European case definitions [3]. Children with lesions <5 cm were also included when the treating clinician judged the appearance and evolution of multiple lesions to be consistent with EM, in keeping with previous pediatric observations [16]. Routine skin biopsy or microbiological confirmation of lesions was not performed. Clinical reassessment at treatment completion offered an opportunity to identify lesions that persisted or developed atypical features; children subsequently given an alternative dermatological diagnosis were excluded. The retrospective records do not permit independent confirmation of every skin lesion.

### 2.2. Data Collection

Data extracted from the medical records included age, month of presentation, history and anatomical location of tick bite, constitutional symptoms before antibiotic therapy, timing, number, distribution and diameter of skin lesions, laboratory findings, initial antibiotic treatment, and documented clinical course. Complete blood counts at the end of treatment were used to describe the availability of end-of-treatment assessment. Follow-up by the referring pediatrician approximately one month later was routinely requested, but those assessments were not systematically available in the study records. Age-specific laboratory reference limits from the Clinical Institute of Clinical Chemistry and Biochemistry, University Medical Centre Ljubljana, were applied to each child when classifying values as outside the reference range (Table S1).

During the study period, lumbar puncture was not routinely performed in children with MEM and was reserved for patients with clinical signs suggestive of neurological involvement. Lyme neuroborreliosis was diagnosed according to institutional criteria [17] and interpreted in relation to established European diagnostic criteria [18].

Tests for TBE virus, *Anaplasma phagocytophilum*, and other tick-borne pathogens were not performed systematically for this retrospective cohort. Testing was undertaken when clinically indicated; the supplementary case summary describes one child who underwent TBE serology (Text S1).

### 2.3. Statistical Analysis

Data were entered into Microsoft Excel and analyzed using descriptive statistics. Continuous variables are presented as medians with interquartile ranges (IQRs), whereas categorical variables are presented as frequencies and percentages. Exact binomial (Clopper–Pearson) 95% confidence intervals (CIs) were calculated for proportions.

### 2.4. Ethics

The study was approved by the National Medical Ethics Committee of the Republic of Slovenia (approval No. 0120-385/2026-2711-3). Owing to the retrospective nature of the study and the use of routinely collected clinical data, the requirement for informed consent was waived.

## 3. Results

### 3.1. Study Population

Between January 2021 and December 2025, 621 children were diagnosed with disseminated Lyme borreliosis at the Department of Infectious Diseases, University Medical Centre Ljubljana. Of these, 445 (72%; 95% CI 68–75%) had MEM and were included in the study. One child was excluded because skin lesions initially diagnosed as MEM were subsequently identified as annular lichenoid dermatitis of youth based on histopathological findings and clinical follow-up.

The annual number of children with MEM ranged from 68 to 102. Two children experienced two separate episodes of MEM during the study period, occurring 13 and 14 months apart, respectively.

### 3.2. Demographic and Seasonal Characteristics

**Figure 1:**
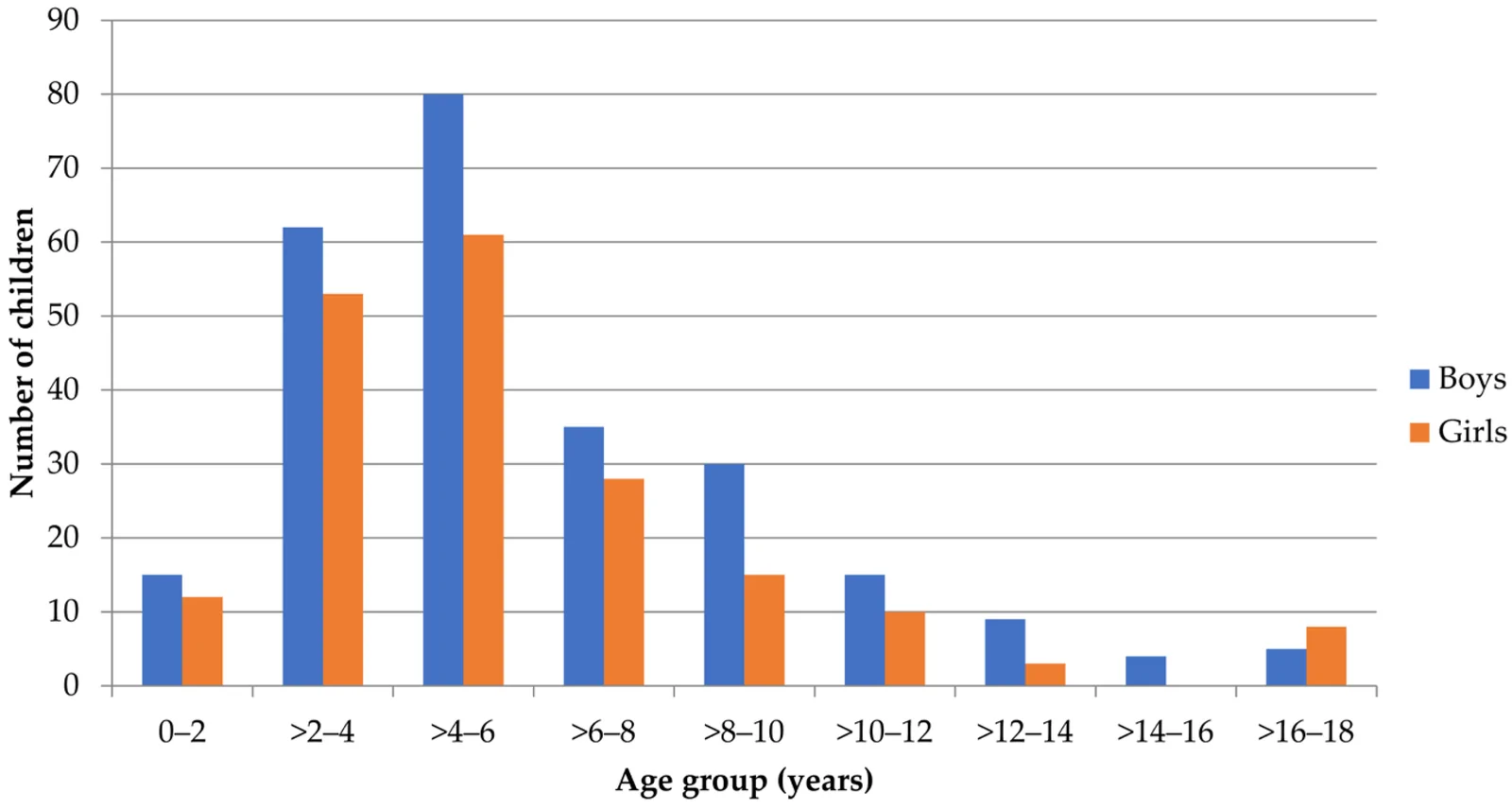
Age distribution of 445 children with multiple erythema migrans according to sex.

Of the 445 children, 255 were boys (57%, 95% CI 53–61%). The median age was 5.0 years (IQR 3.6–7.6; range 0.6–17.5 years). The age distribution was markedly skewed toward younger children, with 348/445 (78%; 95% CI 74–82%) children being younger than 8 years (Figure 1).

Most children presented between May and July (272/445, 61%, 95% CI 56–66%) (Figure S1), indicating a pronounced seasonal distribution of MEM.

### 3.3. Tick Bite Characteristics

Information on a preceding tick bite was available for 429 of 445 children (96%). A tick bite was recalled by 264 children (61.5%; 95% CI 56.8–66.1%), whereas 165 (38.5%; 95% CI 33.8–43.2%) did not recall one.

The date of the tick bite was documented in 179 children. In 148 (83%; 95% CI 76–88%), the bite had occurred within one month before the appearance of the first skin lesion.

Information on the anatomical site of the tick bite was available for 147 children (56% of those recalling a tick bite). The head and neck region was the most frequently reported location (43%; 95% CI 35–51%), followed by the trunk (30%; 95% CI 23–38%), lower extremities (17%; 95% CI 11–24%), and upper extremities (16%; 95% CI 11–23%). Twelve children (8%; 95% CI 4–14%) reported tick bites at more than one anatomical site (Table S2).

### 3.4. Characteristics of Skin Lesions

Information on the interval between the appearance of the initial erythema migrans (EM) lesion and subsequent lesions was available for 194 of 445 children (44%). The median interval was 2 days (IQR 1–5). In 153 children (34%; 95% CI 30–39%), multiple lesions were already present when the rash was first noticed, precluding estimation of the interval between the initial and subsequent lesions.

Twenty-five children initially received oral antibiotics for a lesion considered to represent solitary EM (phenoxymethylpenicillin, n = 14; amoxicillin, n = 6; azithromycin, n = 5) before additional lesions were recognized. The interval from the initial lesion to subsequent lesions was 0–2 days in 11 (44%; 95% CI 24–65%) and more than 14 days in six (24%; 95% CI 9–45%); the median was 4 days (IQR 1.0–13.5). In the available records, additional lesions were first noted on the day oral treatment started or the next day in 16 children, later in seven, and at an unspecified time in two. A number of doses or treatment duration was recorded for six children (one dose in three, three doses in two, and three days in one); adherence was not documented. These timing variables describe different intervals and cannot establish why additional lesions appeared.

The number of EM lesions was recorded in 398 of 445 children (89%). The median number of lesions was 4 (IQR 2–6), with a maximum of 41. Most children had 2–5 lesions (282/398, 71%; 95% CI 66–75%), whereas more than 10 lesions were observed in 55 children (14%; 95% CI 11–18%) (Table 1).

The maximum diameter of an EM lesion was documented in 264 children (59%). The median maximum diameter was 7 cm (IQR 5–10), with a maximum of 30 cm. In 58 of 264 children (22%; 95% CI 17–27%), the largest lesion measured less than 5 cm.

Information on the regional distribution of skin lesions was available for 442 children. Lesions were confined to a single anatomical region in only 41 of 442 children (9%; 95% CI 7–12%).

### 3.5. Constitutional Symptoms

Information on constitutional symptoms was available for 430 children. Of these, 310 (72%; 95% CI 68–76%) reported no constitutional symptoms before antibiotic treatment, whereas 120 (28%; 95% CI 24–32%) reported at least one symptom. Symptom status was unclear for the remaining 15 children (Table 2).

Among the 120 symptomatic children, 66 reported symptoms only before the appearance of the first skin lesion, nine reported symptoms both before and at or after skin-lesion onset, and 45 reported symptoms only at or after skin-lesion onset. Thus, 75 children (63%; 95% CI 53–71%) had symptoms before skin-lesion onset and 54 had symptoms at or after onset; nine children were included in both timing groups. Three children reported symptoms at more than one time point after skin-lesion onset.

Among the 75 children who experienced symptoms before the onset of skin lesions, 35 (47%; 95% CI 35–59%) reported a single symptom. The most frequent isolated symptoms were fever (*n* = 17), pain in the extremities, muscles, or joints (*n* = 6), headache (*n* = 3), fatigue (*n* = 3), gastrointestinal symptoms (*n* = 3), irritability (*n* = 2), and malaise (*n* = 1). Forty of 75 children (53%; 95% CI 41–65%) reported more than one symptom, with fever and headache being the most frequent combination (n = 12).

Among children with available information, symptoms preceding the skin lesions began a median of 8.5 days before the onset of erythema migrans (IQR 3–13.75; n = 58) and lasted for a median of 1.5 days (IQR 1–3; n = 40). Fever and headache were the most frequently reported symptoms both before and after the onset of skin lesions (Table 2). Among children with available data, these symptoms were typically brief and, when they preceded the first skin lesion, occurred several days before its appearance.

#### Extracutaneous Manifestations

Clinically significant extracutaneous manifestations were uncommon. Two children (0.4%; 95% CI 0.05–1.6%) had Lyme neuroborreliosis (Text S1): one presented with peripheral facial nerve palsy and the other developed meningitis during treatment. One additional child had isolated lymphadenopathy.

### 3.6. Laboratory Findings

Routine laboratory test results obtained before initiation of antibiotic therapy were available for most children (Table 3). Median leukocyte, platelet, neutrophil, lymphocyte, eosinophil, aspartate aminotransferase (AST), alanine aminotransferase (ALT), and gamma-glutamyl transferase (GGT) values fell within the illustrative reference ranges for a five-year-old child. Each child’s age-specific limits (Table S1) were used to calculate the proportions with values outside the reference range (Table S3).

The proportions of children with laboratory values outside their age-specific reference ranges were 8% (33/432; 95% CI 5–11%) for leukocyte count, 11% (48/432; 95% CI 8–14%) for platelet count, 4% (19/429; 95% CI 3–7%) for neutrophil count, 5% (22/429; 95% CI 3–8%) for lymphocyte count, 11% (46/429; 95% CI 8–14%) for absolute eosinophil count, 11% (43/400; 95% CI 8–14%) for AST, 7% (28/429; 95% CI 4–9%) for ALT, and 3% (13/429; 95% CI 2–5%) for GGT (Table S3). Overall, most routine laboratory results were within the relevant age-specific intervals.

### 3.7. Treatment and Outcome

Intravenous ceftriaxone was administered to 424 of 445 children (95%). Oral doxycycline and amoxicillin were prescribed in 19 and 2 children, respectively.

An end-of-treatment complete blood count was available for 233 children (52%), reflecting the availability of a clinical assessment around treatment completion, usually on day 14. Information on the time to complete resolution of skin lesions was available for 138 children (31%). The median time from initiation of antibiotic therapy to complete disappearance of skin lesions was 3 days (IQR 2–4). Skin lesions persisted through the 14-day treatment course in five of 138 children (4%; 95% CI 1–8%); the subsequent date of resolution was not documented. Results of the follow-up requested from referring pediatricians approximately one month later were not systematically available.

## 4. Discussion

This large contemporary cohort provides a detailed description of pediatric MEM in a highly endemic region. MEM predominantly affected young children; additional lesions usually appeared within a few days of the initial lesion, constitutional symptoms were absent in nearly three-quarters of patients, and routine laboratory findings were largely unremarkable. These findings characterize pediatric MEM primarily as a disseminated cutaneous manifestation with a generally uncomplicated clinical course.

The age distribution of MEM was strongly skewed toward younger children, with a median age of 5 years and 78% younger than 8 years. Similar findings have been reported in Slovenian children, in whom MEM occurred at a younger age than solitary EM [19]. Children with cutaneous Lyme borreliosis were likewise the youngest group in the recent Swiss cohort, although only a small proportion had MEM [6].

Approximately two-thirds of children recalled a preceding tick bite, most often in the head and neck region. This distribution has long been recognized in pediatric Lyme borreliosis [20] and was also observed in a recent Swiss pediatric cohort [6]. Because the primary EM lesion could not be reliably identified retrospectively, no conclusions can be drawn regarding possible routes of dissemination from the inoculation site.

Most children had 2–5 lesions, with a median of 4, comparable with previous pediatric studies [6,7,19]. Lesions were confined to a single anatomical region in only 9%, closely matching the Austrian pediatric cohort [7]. The interval between the initial and subsequent lesions was usually short, with a median of 2 days, suggesting that cutaneous dissemination generally becomes clinically apparent rapidly. Occasional longer intervals were observed, but retrospective data cannot distinguish delayed recognition from prolonged dissemination or reinfection.

Constitutional symptoms occurred in 28% of children, with fever and headache the most frequent, consistent with previous reports [8,21]. When these symptoms preceded EM, they were generally brief and occurred several days before skin-lesion onset. Routine laboratory findings were largely within age-adjusted reference ranges, suggesting that MEM is usually not accompanied by a marked systemic inflammatory response.

Two children developed a second MEM episode 13 and 14 months after the first, respectively. Similar observations have recently been reported in adults with repeated disseminated Lyme borreliosis [22]. Because episodes before the study period were not systematically recorded, the frequency of recurrent MEM cannot be estimated, but these cases indicate that a previous disseminated episode does not necessarily prevent subsequent reinfection presenting with disseminated disease.

Treatment in this cohort reflects long-standing Slovenian practice: 95% of children received intravenous ceftriaxone. This approach originated from earlier observations of CSF abnormalities, and occasional isolation of *B. burgdorferi* sensu lato from CSF, in neurologically asymptomatic children with MEM [8]. The present study was not designed to reassess those findings because lumbar puncture was not routinely performed. More importantly, the near-universal use of ceftriaxone precludes comparison with oral therapy. Prospective comparative studies are therefore needed to determine whether oral regimens recommended by current international guidelines provide equivalent clinical outcomes while avoiding the burden of intravenous treatment.

A randomized trial in adults with acute disseminated LB without meningitis compared oral doxycycline with intravenous ceftriaxone and found similar clinical efficacy [23]. The study population and treatment regimens differ from those of our pediatric cohort, so this evidence cannot establish treatment equivalence for children with MEM. Our data also cannot estimate outcomes after initial oral treatment: in the 25 children first treated for presumed solitary EM, additional lesions sometimes became apparent within a day of treatment initiation, while information on doses, completion, and adherence was sparse. The appearance of lesions after an antibiotic was started should not be interpreted as antibiotic-induced dissemination or proof of treatment failure.

The short interval between the first and subsequent lesions and the limited frequency of extracutaneous manifestations help characterize the presentation at referral, but the timing is based on caregiver recall and incomplete records. Multiple lesions were already present when the eruption was first noticed in 153 children, making the interval impossible to estimate in those cases. The finding of largely age-appropriate routine laboratory values describes the distribution in this referred cohort, not a diagnostic test for MEM. Clinical follow-up around the end of treatment offered an opportunity to reconsider atypical or persistent lesions, but information on the later course was incomplete.

The strengths of this study include its large cohort of children with MEM and relatively uniform diagnostic and therapeutic practice at a single infectious diseases centre. Clinical reassessment around treatment completion provided an opportunity to reconsider persistent or atypical lesions; one initial MEM diagnosis was revised after histopathology and follow-up. Nevertheless, no routine lesion biopsy or microbiological confirmation was performed, and the inclusion of lesions <5 cm may have introduced diagnostic misclassification. Limitations also include the retrospective design, incomplete documentation of some clinical variables, near-universal ceftriaxone treatment, absence of routine lumbar puncture, and no systematic information from the later outpatient follow-up. Although an end-of-treatment complete blood count was available in 233 children, time to disappearance of skin lesions was recorded in only 138, and the subsequent outcome of five whose lesions persisted through treatment is unknown. These limitations preclude direct comparison of oral and intravenous therapy and assessment of the natural course of orally treated MEM. Other tick-borne infections were not assessed systematically, so coinfection prevalence cannot be inferred and nonspecific symptoms cannot invariably be attributed to LB alone.

## 5. Conclusions

In conclusion, pediatric MEM in this large contemporary cohort predominantly affected young children and usually followed an uncomplicated clinical course, with rapid development of additional skin lesions, limited constitutional symptoms, and largely normal routine laboratory findings. The study provides updated descriptive data on pediatric MEM but cannot determine the optimal antibiotic regimen because almost all children received ceftriaxone. Prospective comparative studies of oral and intravenous treatment are warranted.

## Figures and Tables

**Table 1:** Number of Erythema Migrans Lesions in Children with Multiple Erythema Migrans (N = 398).

Number of Lesions	No. (%; 95% CI)
2	105 (26.4; 22–31)
3–5	177 (44.5; 40–50)
6–10	61 (15.3; 12–19)
11–20	40 (10.1; 7–13)
21–30	13 (3.3; 2–6)
31–41	2 (0.5; 0–2)

**Table 2:** Constitutional Symptoms in Children with Multiple Erythema Migrans by Timing of Symptom Onset.

Symptom	Before Skin-Lesion Onset, No. (%) (N = 75)	At or After Skin-Lesion Onset, No. (%) (N = 54)
Fever	40 (53.3)	20 (37.0)
Headache	25 (33.3)	15 (27.8)
Fatigue	14 (18.7)	10 (18.5)
Myalgia	10 (13.3)	4 (7.4)
Nausea or vomiting	7 (9.3)	4 (7.4)
Arthralgia	5 (6.7)	2 (3.7)
Diarrhea	5 (6.7)	2 (3.7)
Malaise	4 (5.3)	1 (1.9)
Nasal discharge or cough	4 (5.3)	3 (5.6)
Irritability	4 (5.3)	2 (3.7)
Loss of appetite	3 (4.0)	0
Abdominal pain	2 (2.7)	2 (3.7)
Paresthesia of the toes	1 (1.3)	0
Dizziness	0	2 (3.7)
Conjunctivitis and cough	0	1 (1.9)
Sleep myoclonus	0	1 (1.9)
Sore throat	0	1 (1.9)
Scarlatiniform rash	0	1 (1.9)

Individual children could report more than 1 symptom. Timing groups were not mutually exclusive; values are therefore presented descriptively without between-group hypothesis testing.

**Table 3:** Laboratory Findings in Children with Multiple Erythema Migrans before Antibiotic Therapy.

Parameter	N	Median (IQR)	Range
Leukocytes (×10^9^/L)	432	7.9 (6.5–9.4)	2.8–23.3
Platelets (×10^9^/L)	432	315 (280–366)	135–669
Neutrophils (×10^9^/L)	429	3.6 (2.9–4.8)	0.2–17.5
Lymphocytes (×10^9^/L)	429	3.1 (2.4–3.8)	0.5–10.3
Eosinophils (×10^9^/L)	429	0.2 (0.1–0.4)	0–3.4
AST (µkat/L)	400	0.60 (0.54–0.70)	0.18–2.57
ALT (µkat/L)	429	0.34 (0.29–0.42)	0.13–4.12
GGT (µkat/L)	429	0.22 (0.18–0.26)	0.02–1.48

ALT indicates alanine aminotransferase; AST, aspartate aminotransferase; GGT, gamma-glutamyl transferase; IQR, interquartile range. Illustrative reference intervals for a five-year-old child (the cohort median age) are: leukocytes 5.1–12.9 × 10^9^/L; platelets 150–410 × 10^9^/L; neutrophils 1.7–8.5 × 10^9^/L; lymphocytes 1.8–7.0 × 10^9^/L; eosinophils 0.02–0.75 × 10^9^/L; AST < 0.78 µkat/L; ALT < 0.68 µkat/L; GGT < 0.53 µkat/L. The age-specific limits used for individual children are shown in Table S1.

## Data Availability

The original contributions presented in this study are included in the article/Supplementary Material. Further inquiries can be directed to the corresponding author.

## Supplementary Materials

The following supporting information can be downloaded at https://www.mdpi.com/article/10.3390/pathogens15101037/s1, Figure S1: Monthly distribution of 445 children with multiple erythema migrans; Table S1: Age-adjusted reference values for blood test results; Text S1: Clinical summaries of 2 children with multiple erythema migrans and Lyme neuroborreliosis; Table S2: Anatomical location of reported tick bites in children with multiple erythema migrans; Table S3: Laboratory values outside age-based reference ranges in children with multiple erythema migrans.

## Abbreviations

The following abbreviations are used in this manuscript:

LB	Lyme borreliosis
EM	Erythema migrans
MEM	Multiple erythema migrans
TBE	Tick-borne encephalitis
CSF	Cerebrospinal fluid
IQR	Interquartile range
CI	Confidence interval
