# Supplementary material for: Clinical Characteristics of Multiple Erythema Migrans in Children: A Five-Year Retrospective Study
Source: Pathogens. 2026 Oct 1;15(10):1037. doi: 10.3390/pathogens15101037 (PMC13708218; doi:10.3390/pathogens15101037)
Supplement: Supplementary file 1 [file pathogens-15-01037-s001.zip › pathogens-4586138-supplementary.pdf]

Supplementary Materials

Figure S1: Monthly distribution of 445 children with multiple erythema migrans.

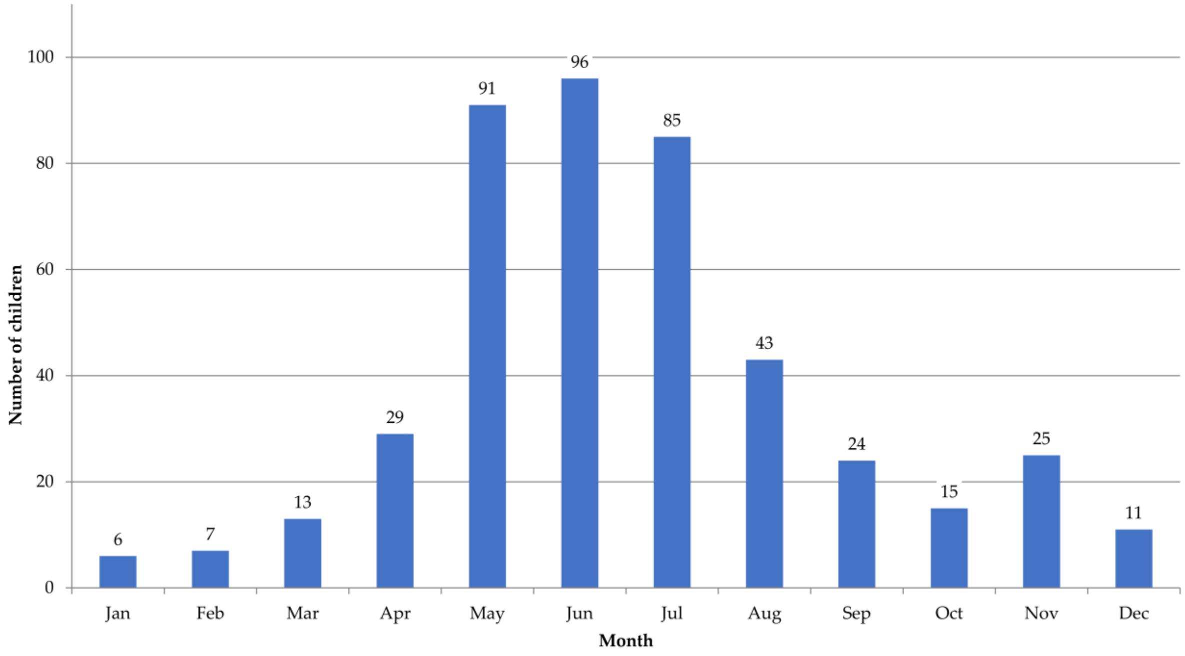

Table S1: Age-adjusted reference values for blood test results.

**Laboratory parameter / Age**	**< 1**	**1**	**2**	**3**	**4**	**5**	**6**	**7**	**8**	**9**	**10**	**11**	**12**	**13**	**14**	**15**	**16**	**17**
Leukocytes	6,6	6	5,4	5,4	5,1	5,1	4,8	4,8	4,8	4,8	4,8	4,8	4,5	4,5	4,5	4,2	4,2	4,2
	15,6	15	13,8	13,8	12,9	12,9	12	12	12	12	12	12	11,4	11,4	11,4	10,8	10,8	10,8
Platelets	150	150	150	150	150	150	150	150	150	150	150	150	150	150	150	150	150	150
	410	410	410	410	410	410	410	410	410	410	410	410	410	410	410	410	410	410
Neutrophils	1,5	1,50	1,5	1,5	1,7	1,7	1,7	1,7	1,7	1,7	1,7	1,7	1,7	1,7	1,7	1,7	1,7	1,7
	8,7	8,7	8,5	8,5	8,5	8,5	8,1	8,1	8,1	8,1	8,1	8,1	7,9	7,9	7,9	7,9	7,9	7,9
Lymphocytes	3,2	3	2,2	2,2	1,8	1,8	1,5	1,5	1,5	1,5	1,5	1,5	1,2	1,2	1,2	1,2	1,2	1,2
	11,2	10	8,5	8,5	7	7	6	6	6	6	6	6	5	5	5	5	5	5
Eosinophils	0,05	0,03	0,02	0,02	0,02	0,02	0,02	0,02	0,02	0,02	0,02	0,02	0,02	0,02	0,02	0,02	0,02	0,02
	0,8	0,7	0,75	0,75	0,75	0,75	0,7	0,7	0,7	0,7	0,7	0,7	0,65	0,65	0,65	0,55	0,55	0,55
Eosinophils %	0,5	0,5	0,5	0,5	0,5	0,5	0,5	0,5	0,5	0,5	0,5	0,5	0,5	0,5	0,5	0,5	0,5	0,5
	5	5	5	5	5,5	5,5	5,5	5,5	5,5	5,5	5,5	5,5	5,5	5,5	5,5	5,5	5,5	5,5
AST	<1,22	<0,86	<0,86	<0,86	<0,78	<0,78	<0,78	<0,69	<0,69	<0,69	<0,69	<0,69	<0,69	<0,62	<0,62	<0,62	<0,61	<0,61
ALT	<0,98	<0,68	<0,68	<0,68	<0,68	<0,68	<0,68	<0,68	<0,68	<0,68	<0,68	<0,68	<0,48	<0,48	<0,48	<0,48	<0,48	<0,48
GGT	<0,53	<0,53	<0,53	<0,53	<0,53	<0,53	<0,53	<0,53	<0,53	<0,53	<0,53	<0,53	<0,8	<0,58	<0,58	<0,58	<0,8	<0,58

The upper and lower limits for blood test results are stated by age. ALT indicates alanine aminotransferase; AST, aspartate aminotransferase; GGT, gamma-glutamyl transferase.

Age is expressed in years. Leukocyte, platelet, neutrophil, lymphocyte and eosinophil values are expressed in $\times 10^9$/L. AST, ALT and GGT values are expressed in µkat/L.

Source of reference values: Clinical Institute of Clinical Chemistry and Biochemistry, University Medical Centre Ljubljana.

Text S1: Clinical summaries of 2 children with multiple erythema migrans and Lyme neuroborreliosis

Case 1. A 6-year-old boy presented in June with 4 EM lesions 2 months after a recalled tick bite. Seven days before the first lesion, he had a 2-day febrile upper respiratory illness. Ceftriaxone was started at presentation. On treatment day 3, he developed headache and vomiting; several days later, neck stiffness was noted. CSF examination showed pleocytosis (55 cells/μL), protein 0.24 g/L, and intrathecal production of Borrelia-specific IgM. CSF enterovirus PCR and tick-borne encephalitis serology were negative. Blood and CSF Borrelia cultures were negative. He completed 14 days of ceftriaxone. Symptoms resolved, and no neurological complaints were reported at 3-month follow-up.

Case 2. A 9-year-old girl presented with right peripheral facial nerve palsy, headache, low-grade fever, and 3 EM lesions. Meningeal signs were absent. Because facial palsy with EM met institutional criteria for LNB, lumbar puncture was not performed. She received 14 days of ceftriaxone together with eye-protection measures and physiotherapy. Skin lesions resolved during treatment, and facial symmetry was almost normal at 3-month follow-up.

Table S2: Anatomical location of reported tick bites in children with multiple erythema migrans.

**Body region**	**No. (%; 95% CI)**
Head and neck	63 (43; 35—51)
Trunk	44 (30; 23—38)
Upper extremity	24 (16; 11—23)
Lower extremity	25 (17; 11—24)

Children could report tick bites at more than 1 anatomical site; percentages therefore sum to >100%.

Table S3: Laboratory values outside age-adjusted reference ranges in children with multiple erythema migrans.

Laboratory parameter	Children with value outside reference range, No./N (%; 95%CI)
Leukocytes elevated for age	17/432 (4; 2—6)
Leukocytes lowered for age	16/432 (4; 2—6)
Platelets $< 150 \times 10^9$/L	1/432 (0.2; 0—1)
Platelets $> 410 \times 10^9$/L	47/432 (11; 8—14)
Neutrophils lowered for age	19/429 (4; 3—7)
Lymphocytes lowered for age	20/429 (5; 3—7)
Lymphocytes elevated for age	2/429 (0.5; 0.1—1.7)
% eosinophils elevated for age	80/429 (18; 15—23)
Eosinophils elevated for age	30/429 (7; 5—10)
Eosinophils lowered for age	16/429 (4; 2—6)
AST elevated for age	43/400 (11; 8—14)
ALT elevated for age	28/429 (7; 4—9)
GGT elevated for age	13/429 (3; 2—5)

ALT indicates alanine aminotransferase; AST, aspartate aminotransferase; GGT, gamma-glutamyl transferase.
